# Prediction model for knee osteoarthritis based on genetic and clinical information

**DOI:** 10.1186/ar3157

**Published:** 2010-10-12

**Authors:** Hiroshi Takahashi, Masahiro Nakajima, Kouichi Ozaki, Toshihiro Tanaka, Naoyuki Kamatani, Shiro Ikegawa

**Affiliations:** 1 Laboratory for Statistical Analysis, Center for Genomic Medicine, RIKEN, 4-6-1 Shirokanedai, Minato-ku, Tokyo 108-8639, Japan; 2 Laboratory for Bone and Joint Diseases, Center for Genomic Medicine, RIKEN, 4-6-1 Shirokanedai, Minato-ku, Tokyo 108-8639, Japan; 3 Laboratory for Cardiovascular Diseases, Center for Genomic Medicine, RIKEN, 1-7-22 Suehiro-cho, Tsurumi-ku, Yokohama, Kanagawa 230-0045, Japan

## Abstract

**Introduction:**

Osteoarthritis (OA) is the most common bone and joint disease influenced by genetic and environmental factors. Recent association studies have uncovered the genetic factors behind OA, its susceptibility genes, which would enable us to predict disease occurrence based on genotype information. However, most previous studies have evaluated the effects of only a single susceptibility gene, and hence prediction based on such information is not as reliable. Here, we constructed OA-prediction models based on genotype information from a case-control association study and tested their predictability.

**Methods:**

We genotyped risk alleles of the three susceptibility genes, asporin (*ASPN*), growth differentiation factor 5 (*GDF5*), and double von Willebrand factor A domains (*DVWA*) for a total of 2,158 Japanese subjects (933 OA and 1,225 controls) and statistically analyzed their effects. After that, we constructed prediction models by using the logistic regression analysis.

**Results:**

When the effects of each allele were assumed to be the same and multiplicative, each additional risk allele increased the odds ratio (OR) by a factor of 1.23 (95% confidence interval (CI), 1.12 to 1.34). Individuals with five or six risk alleles showed significantly higher susceptibility when compared with those with zero or one, with an OR of 2.67 (95% CI, 1.46 to 4.87; *P *= 0.0020). Statistical evaluation of the prediction power of models showed that a model using only genotyping data had poor predictability. We obtained a model with good predictability by incorporating clinical data, which was further improved by rigorous age adjustment.

**Conclusions:**

Our results showed that consideration of adjusted clinical information, as well as increases in the number of risk alleles to be integrated, is critical for OA prediction by using data from case-control studies. To the authors' knowledge, this is the first report of the OA-prediction model combining both genetic and clinical information.

## Introduction

Osteoarthritis (OA) is the most common bone and joint disease and is characterized by progressive cartilage degeneration. OA is a polygenic disease caused by genetic and environmental factors [1]. Epidemiologic studies have suggested that genetic factors strongly affect the onset and development of OA [2]. Genetic association studies are now uncovering the genetic factors responsible for of OA, that is, its susceptibility genes. Candidate-gene approaches have identified several genes associated with OA, and genome-wide association studies have recently found several promising OA-susceptibility genes [1,3].

Identification of OA-susceptibility genes would enable us to predict disease phenotypes based on genotype information. Such predictions would be a basis for personalized medicine and disease prevention. However, most previous OA genetic studies have evaluated the effects of only single susceptibility genes, which are mostly small, and hence predictions based on their results are not always useful. By analyzing the combinatorial effects of the gene calmodulin 1 (*CALM1*) and other genes in hip OA susceptibility, combinatorial association between *CALM1 *and *ASPN *susceptibility alleles has been reported [4]; however, the association was not conclusive because of the small sample size of the study. Because OA is a polygenic disease, evaluation of the combined effects of susceptibility genes, as well as between susceptibility genes and environmental/clinical factors, is important.

To obtain a more-comprehensive view of knee OA susceptibility and to make a good prediction model for OA by using genotype information, we investigated the combined effects of known susceptibility genes for knee OA in Japanese persons. We examined gene-gene interactions of the previously reported OA genes and estimated the population attributed risk (PAR) statistic of the genes by using data of a case-control association study [5]. We constructed prediction models for OA by using genetic data only and a combination of genetic and clinical data, and evaluated their predictability statistically. The model using only genotype information had less than fair predictability, but integration of clinical data with adjustment showed marked improvement of the prediction power.

## Materials and methods

### Subjects

A case-control cohort (692 cases and 748 controls) was recruited from patients of several medical institutes in Japan. A population-based cohort (241 cases and 477 controls) was recruited from inhabitants of Odai and Minami-ise town in the Mie prefecture in Japan. We obtained written informed consent from each subject, and the study was approved by the ethics committee of the Center for Genomic Medicine in RIKEN. In total, 933 knee OA (81.1% female; mean age ± standard deviation (SD) = 71.8 ± 7.7 years) and 1,225 control subjects (75.4% female; mean age ± SD = 69.3 ± 9.2 years) were included. OA was diagnosed based on clinical and radiographic findings by using previously described criteria [6]. All OA patients were older than 40 years.

### Genotyping

DNA was extracted from peripheral blood by using standard methods. *ASPN *D-repeat polymorphism [7], rs143383 in *GDF5 *[8], and rs7639618 in *DVWA *[6] were genotyped as described previously.

### Statistical analysis

We used the software R [9] for all statistical analyses. To obtain PAR for multiple risk factors among case-control study data, we used an OR assessed by the logistic regression model adjusted for gender and age [10].

### Prediction model

We used logistic regression for constructing prediction models. This method assumes a multiplicative allelic effect (each allele independently increases the odds of the disease). We used the Cochran-Armitage trend test to examine this odds increase by increasing the number of risk alleles. The variables of the logistic regression model with clinical data remained after the stepwise selection of variables based on the likelihood ratio and the Wald statistics [11,12]. We used the receiver operating characteristic (ROC) curve to evaluate abilities of prediction models. The area under the curve (AUC), which is a measure of the power to distinguish case and control individuals, was calculated for the curve by using the ROCR package [13]. The simulation study for the adjusted control group was performed 1,000 times. A perfect model would have an AUC of 1, whereas a model with no discriminative power would have an AUC of 0.5 [14].

### Estimation of contribution of the genetic factor

We considered a statistical model for prediction as follows:

Phenotype (P)=Genotype (G)+Clinical factor (E).

By taking variances on the liability scale for both sides of equation, this model becomes:

Var(P)=Var(G)+Var(E)+Cov(G,E).

As we found no evidence of gene-environmental interactions, we could assume that *Cov*(*G, E*) = 0. We also estimated the contribution of genetic factors in our models by calculating the average proportion of variation, *Var*(*E*)/*Var*(*P*), for 1,000 simulation studies.

## Results

### Selection of susceptibility genes

We selected three susceptibility genes, *ASPN *[7], *GDF5 *[8], and *DVWA *[6], for study because they had clear associations in Japanese subjects and because the associations were replicated in other ethnic populations and supported by functional evidence for OA causality [1,3]. We obtained clinical data (Table 1) and genotyping data (Table 2) for 933 knee OA cases and 1,225 controls and examined whether the previously reported risk alleles were associated with knee OA in this population after genotyping. We observed significant associations in *GDF5 *and *DVWA *and marginal associations in *ASPN *(Table 3).

**Table 1:** Clinical data of subjects

Variable	Case		Control	
		
	Female	Male	Female	Male
Number (% of subjects)	757 (81.1)	176 (18.9)	924 (75.4)	301 (24.6)
Age, years (SD)	71.6 (7.5)	72.9 (8.7)	70.3 (8.7)	66.2 (10.2)
Height, cm (SD)	150.2 (6.1)	161.5 (6.8)	149.9 (6.1)	162.9 (7.5)
Weight, kg (SD)	56.2 (8.9)	63.8 (11.1)	51.5 (9.0)	63.8 (10.7)
BMI, kg/m^2 ^(SD)	24.9 (12.7)	24.4 (11.0)	22.9 (12.1)	23.9 (8.75)

Only subjects successfully genotyped for all polymorphisms are included. SD, standard deviation.

**Table 2:** Genotyping data of subjects

Gene		**Genotype count **^**a**^			Risk-allele frequency
			
		Homo	Hetero	Others	
*ASPN*	Case	9	127	797	0.078
	Control	3	147	1,075	0.062
*GDF5*	Case	566	313	54	0.774
	Control	684	461	80	0.747
*DVWA*	Case	369	428	136	0.625
	Control	397	600	228	0.569

^a^For the risk allele. ASPN, aspirin; DVWA, double von Willebrand factor A domain; GDF5, growth differentiation factor 5.

**Table 3:** Association between three genes and knee OA

Gene	Odds ratio	95% CI	***P *value**^**a**^
*ASPN*	1.26	1.00 to 1.60	0.058
*GDF5*	1.17	1.01 to 1.34	0.037
*DVWA*	1.26	1.12 to 1.43	0.0024

^a^*P *values based on χ^2 ^association test to 2 × 2 tables by allele model for *GDF5 *and *DVWA*, and by D14 versus others for ASPN. ASPN, aspirin; CI, confidence interval; DVWA, double von Willebrand factor A domain; GDF5, growth differentiation factor 5.

### Association between number of risk alleles and knee OA

We tested the independence between risk alleles for all pair-wise combinations by a χ^2 ^association test to 3 × 3 tables by using both case-only and case-control mixed designs. We did not find evidence of dependence. We next examined gene-gene interactions by using the logistic regression model. We considered analysis of variance between two logistic regression models, with or without the term of gene-gene interaction. After Bonferroni correction (*P *= 0.05/3 for three pair-wise combinations among three genes), we did not find any evidence of interaction (Table 4).

**Table 4:** Test of independence and gene-gene interactions

Interaction	*P *value		
	
	**Case-control**^**a**^	**Case-only**^**b**^	**Two models**^**c**^
*ASPN *× *GDF5*	0.598	0.273	0.056
*GDF5 *× *DVWA*	0.416	0.701	0.920
*DVWA *× *ASPN*	0.197	0.104	0.290

^a, b^*P *values based on χ^2 ^association test to 3 × 3 tables by genotype model. ^c^ANOVA for two logistic regression models, with or without interactions. ASPN, aspirin; DVWA, double von Willebrand factor A domain; GDF5, growth differentiation factor 5.

**Figure 1:**
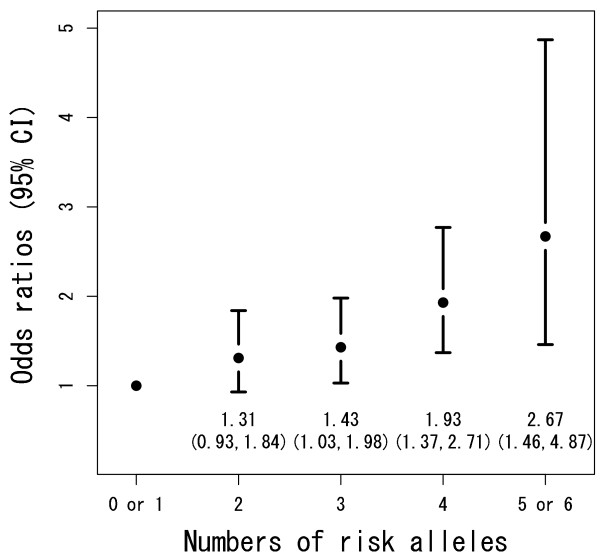
**Odds ratios for subjects with different numbers of risk alleles for knee OA**. Effects of the number of *ASPN*, *GDF5*, and *DVWA *susceptibility risk alleles on knee osteoarthritis (OA) risk. Odds ratios (ORs) and their 95% confidence intervals according to the number of risk alleles were calculated by using a logistic regression model adjusted for gender, age, and body mass index. We set the control group as subjects who have zero or one risk allele. Each additional risk allele increased the OR by 1.22 (95% CI, 1.11 to 1.34), which indicated the cumulative effects of the three alleles on knee OA.

We then studied the association between knee OA and the number of risk alleles possessed by a subject (Table 5). The distributions of the subjects over the number of risk alleles differed between the case and control groups. The odds ratio adjusted for age, gender, and body mass index (BMI) increased with the number of risk alleles (Figure 1) (*P *= 4.15e-6). As only 0.7% of subjects in the control group carried zero risk alleles, we selected subjects with either zero or one risk allele as a reference (the lowest-risk) group. The proportion of the group was 10.6%. Similarly, we combined subjects with five or six risk alleles to construct the highest-risk group. The group had an OR of 2.67 (95% CI, 1.46 to 4.87; *P *= 0.0020), as compared with the reference group.

**Table 5:** Number of risk alleles possessed by subjects

Allele	0	1	2	3	4	5	6
Case	6	61	224	357	252	30	3
Control	9	121	332	485	254	24	0

### Estimation of PAR

To investigate the contribution of genetic factors for the onset OA, we estimated the PAR statistic [5]. PAR is the proportion of cases in the population that can avoid the disease if certain risk alleles are removed from the population. Hence, PAR is useful for providing a measure of how much a certain factor contributes to the disease. We compared the reference group with other groups and obtained an estimated PAR of 31.4% for the risk alleles of the three susceptibility genes. We also considered PAR for BMI because the Framingham study indicated that a higher BMI may increase the OA risk [2]. We classified BMIs for all subjects into three categories (normal/underweight, <25; overweight, 26 to 30, and obese, ≥30) [15]. Setting normal/underweight as a reference group, we estimated the PAR of BMI as 28.4%.

### Application for prediction of OA susceptibility

We generated OA-prediction models by using genetic or clinical data or both. We first examined the interactions between genetic and clinical factors and found no evidence of interactions, or of gene-gene interactions. We next considered two logistic regression models: the first model (MODEL I) was constructed with only the number of risk alleles for the three susceptibility genes; the second model (MODEL II) incorporated the clinical information of individuals, including gender, age, and BMI. For both models, we confirmed that each additional risk allele increased ORs by 1.23 (95%CI, 1.12 to 1.34) and 1.22 (95%CI, 1.11 to 1.34), respectively. For MODEL II, the contributions of gender, age, and BMI were 1.35 for female, 1.05 per year older, and 1.18 per unit increase in BMI, respectively.

By a logit transformation, the probability induced by MODEL I is expressed as

$$P=\frac{\exp(0.856+0.205\times n)}{1+\exp(0.856+0.205\times n)}$$

where *n *is the number of risk alleles.

**Figure 2:**
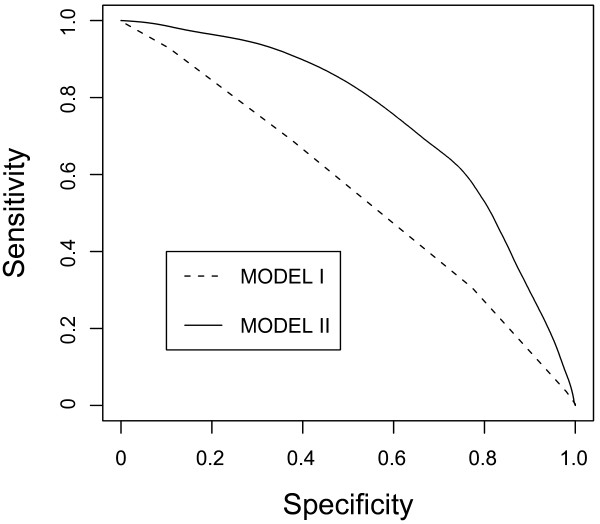
**ROC (receiver operating characteristic) curves of two prediction models**. ROC curves of prediction models that used only genetic factors (MODEL I, AUC = 0.554) and that used both genetic factors and clinical data with age adjustment in the selected control group (MODEL II, AUC = 0.742). The curve for MODEL II was drawn through 1,000 simulation studies with rigorous age adjustment for control groups that reflected the Japanese elderly population. MODEL II had a significantly improved AUC. Curves were described by using the ROCR package.

In MODEL II, the probability is expressed with clinical data of the subjects as

$$P=\frac{\exp(-8.395+0.200\times n+0.302\times gen+0.045\times age+0.162\times BMI)}{1+\exp(-8.395+0.200\times n+0.302\times gen+0.045\times age+0.162\times BMI)}$$

where the value of *gen *is 1 or 2 if the subject is male or female, respectively. We drew an ROC curve and evaluated the predictive power of the model by AUC [16]. AUCs of MODEL I and MODEL II were 0.554 and 0.685, respectively (Figure 2).

To examine the contribution of clinical factors, we constructed a model by using only clinical data (MODEL III). We obtained an AUC of 0.678, which was very similar to that of MODEL II; however, comparison between MODEL II and MODEL III by a likelihood ratio test showed a significant difference (*P *= 1.67e-5), indicating that MODEL II was superior to MODEL III.

To examine the validity of the prediction model, we did a validation study as follows: We divided our subjects into two sets, the cohort study (718 subjects) and the others (1,440 subjects). By using data of the latter set, we constructed MODEL II and applied the model to the data of the former set. Consequently, we obtained an AUC of 0.717 for the validation study, whereas the AUC for the original study was 0.649.

### Adjustment of clinical data for the control group

A prediction model is generally interpreted to be excellent, good, or fair, when its AUC is 0.9 to 1.0, 0.8 to 0.9, or 0.7 to 0.8, respectively [17]. Therefore, our models were poor. This was partly because the proportion of male subjects in our control group was only 0.246, which does not reflect that of the general Japanese population. Hence, we adjusted the age by selecting subjects from the case and control groups whose ages were 60 years or older for constructing MODEL II. Mean ages ± SD (years) of the case and control groups were 73.2 ± 6.2 and 72.0 ± 6.8, respectively. We then constructed a control group with random sampling for 171 male and 215 female sunjects, which was the same male-to-female ratio as that in the Japanese population in 2005. We also selected 400 case subjects at random and applied the logistic regression model to them. Through the simulation studies, we obtained a mean AUC of 0.867.

The distribution of subject ages in our control group was slightly different from that of the general Japanese population. Thus, we selected 128 male and 163 female sunjects so that the subjects in the control group had an equivalent age distribution to the Japanese population in 2005. We then applied the logistic regression model to 291 randomly selected cases. The simulation studies showed a mean AUC of 0.742 for MODEL II (Figure 2). For the model, we estimated the contribution of genetic factors by calculating the proportion of variation and found it very low (0.060), which indicated a nonsignificant contribution of the genetic factor in the model. Actually, we obtained a mean AUC of 0.735 for MODEL III. To confirm the prediction models, we also performed cross-validation studies and obtained their AUCs as 0.677 and 0.671, respectively, which were not different from the original ones.

Our case subjects mainly consisted of women (81.1%), and epidemiology studies have reported that women have a higher risk of OA than do men [2]. Hence, we also applied our models to only male or female subjects. With age adjustment for control subjects, we used 128 cases and 128 controls for men, and 462 cases and 462 controls for women. For men and women, we obtained AUCs of 0.737 and 0.696, respectively, and estimated the contribution of the genetic factor to be 0.110 and 0.091, respectively. The increasing proportions of genetic factors in these models were attributed to the fact that they did not contain gender information.

## Discussion

Classic twin studies on OA heritability suggested that more than half of variations related to the OA susceptibility can be explained by genetic factors [18]. Therefore, we first tested a model that considered genotype information only. The prediction model had a low predictability; its AUC was only 0.554. The insufficient power of the model was partly due to the small number of integrated risk alleles. It has been reported that 20 to 25 risk alleles with frequencies greater than 0.1 and OR values of 1.5 are required for obtaining an AUC of about 0.8 [19]. A precedent study presented a similar model to our MODEL I and reported the additive effect of candidate genes associated with OA [20].

Based on our PAR analysis, the contributions of genetic factors for OA composed by three risk alleles was similar to that of the BMI (31.4% versus 28.4%). The proportion of variations and estimated contributions of genetic factors for our prediction model was calculated to be very low when compared with the previous twin studies [18]. Finding additional risk variants and integrating them into the model is necessary to increase the power of the model.

To improve the predictability of the model, we integrated clinical information, which increased the AUC to 0.685. We further modified the model by adjusting the age because our control group was composed of older subjects, as compared with the case group. After this adjustment, the AUC increased to 0.742. The effect of age difference between the case and control groups was confirmed by simulating the control group and a random sampling that considered only the male-to-female ratio and selected case subjects under the same conditions. The applied logistic regression model produced an AUC of 0.867. These results showed that despite slight differences in age between the case and control groups, mean values could be inflated. Thus, the distribution of age in the control group was influential for constructing a prediction model, underscoring the importance of age adjustment for the control group.

Among the three susceptibility genes, we found no evidence of interaction. We also found no evidence of interaction between the four clinical factors. We are uncertain whether the lack of interaction is true for only the factors that we examined, but it may be due to the limitations in the power of our study resulting from the sample size. It was previously shown that *GDF5 *may contribute to the variation in height with an estimated additive effect [21]; however, we did not find such an association in our study. Both gene-gene and gene-environment interactions should play roles in common diseases, and we may therefore be able to increase the predictive power by finding the yet-unidentified variants that interact with clinical-environmental factors.

Our prediction model for knee OA was constructed based on data from a case-control association study. In type 2 diabetes, some prediction models have been considered based on case-control study data that have incorporated the number of risk alleles and clinical data [17,22,23]. These approaches have achieved successful outcomes; however, unlike a cohort study, data from control subjects may not reflect the true distribution of ages for the target population. In the case in which susceptibility to disease varies with age, it is necessary to consider the age distribution among the control group. To our knowledge, our approach with adjustment of clinical information is the first to construct prediction models using case-control studies, while considering this problem. Our approach can compare prediction models induced by genetic data, clinical data, and both of them and can estimate the contribution of genetic factors for the last model. It would be useful in future preventive measures against not only OA, but also other common polygenic diseases.

## Conclusions

To our knowledge, this study is the first report of an OA-prediction model combining with genetic and clinical information from a case-control association study. Our prediction model using genotype information from three susceptibility genes had poor predictability; however, predictability improved significantly by incorporating clinical data and by adjusting those data. In the current model, the contribution of genetic factors is small. The identification of more OA-risk polymorphisms is necessary; these should be integrated to achieve better prediction.

## Abbreviations

ASPN: aspirin; AUC: area under the curve; BMI: body mass index; CALM1: calmodulin 1; CI: confidence interval; DVWA: double von Willebrand factor A domain; GDF5: growth differentiation factor 5; OA: osteoarthritis; OR: odds ratio; PAR: population-attributed risk; ROC: receiver operating characteristic; SD: standard deviation.

## Competing interests

The authors declare that they have no competing interests.

## Authors' contributions

HT analyzed and interpreted genotyping data and prepared the manuscript. MN, KO, and TT evaluated the patients, and MN genotyped these samples. NK and SI supervised the whole study. All authors contributed to and read and approved the final manuscript.

## References

[B1] Ikegawa S New gene associations in osteoarthritis: what do they provide, and where are we going? Curr Opin Rheumatol 2007 19 429 434 10.1097/BOR.0b013e32825b079d 17762607

[B2] Felson DT Zhang Y Hannan MT Naimark A Weissman B Aliabadi P Levy D Risk factors for incidence of radiographic knee osteoarthritis in the elderly: the Framingham Study Arthritis Rheum 1997 40 728 733 10.1002/art.1780400420 9125257

[B3] Dai J Ikegawa S Recent advances in association studies of osteoarthritis susceptibility genes J Hum Genet 2010 55 77 80 10.1038/jhg.2009.137 20075947

[B4] Mototani H Mabuchi A Saito S Fujioka M Iida A Takatori Y Kotani A Kubo T Nakamura K Sekine A Murakami Y Tsunoda T Notoya K Nakamura Y Ikegawa S A functional single nucleotide polymorphism in the core promoter region of CALM1 is associated with hip osteoarthritis in Japanese Hum Mol Genet 2005 14 1009 1017 10.1093/hmg/ddi093 15746150

[B5] Benichou J A review of adjusted estimators of attributable risk Stat Methods Med Res 2001 10 195 216 10.1191/096228001680195157 11446148

[B6] Miyamoto Y Shi D Nakajima M Ozaki K Sudo A Kotani A Uchida A Tanaka T Fukui N Tsunoda T Takahashi A Nakamura Y Jiang Q Ikegawa S Common variants in DVWA on chromosome 3p24.3 are associated with susceptibility to knee osteoarthritis Nat Genet 2008 40 994 998 10.1038/ng.176 18622395

[B7] Kizawa H Kou I Iida A Sudo A Miyamoto Y Fukuda A Mabuchi A Kotani A Kawakami A Yamamoto S Uchida A Nakamura K Notoya K Nakamura Y Ikegawa S An aspartic acid repeat polymorphism in asporin inhibits chondrogenesis and increases susceptibility to osteoarthritis Nat Genet 2005 37 138 144 10.1038/ng1496 15640800

[B8] Miyamoto Y Mabuchi A Shi D Kubo T Takatori Y Saito S Fujioka M Sudo A Uchida A Yamamoto S Ozaki K Takigawa M Tanaka T Nakamura Y Jiang Q Ikegawa S A functional polymorphism in the 5' UTR of GDF5 is associated with susceptibility to osteoarthritis Nat Genet 2007 39 529 533 10.1038/2005 17384641

[B9] The R Project for Statistical Computing http://www.r-project.org/

[B10] Bruzzi P Green SB Byar DP Brinton LA Schairer C Estimating the population attributable risk for multiple risk factors using case-control data Am J Epidemiol 1985 122 904 914 4050778 10.1093/oxfordjournals.aje.a114174

[B11] Tango T Takagi H Yamaoka K Logistic Regression Analysis 1996 Tokyo: Asakura Shoten (in Japanese)

[B12] Dobson AJ Barnett AG An Introduction to Generalized Linear Models 2008 Third Boca Raton: CRC Press

[B13] Sing T Sander O Beerenwinkel N Lengauer T ROCR: visualizing classifier performance in R Bioinformatics 2005 21 3940 3941 10.1093/bioinformatics/bti623 16096348

[B14] Hanley JA McNeil BJ The meaning of and use of the area under a receiver operating characteristic (ROC) curve Radiology 1982 143 29 36 7063747 10.1148/radiology.143.1.7063747

[B15] Dal Grande E Gill T Wyatt L Chittleborough CR Phillips PJ Taylor AW Population attributable risk (PAR) of overweight and obesity on chronic diseases: South Australian representative, cross-sectional data, 2004-2006 Obes Res Clin Pract 2009 3 159 168 10.1016/j.orcp.2009.03.004 24345586

[B16] Zweig MH Campbell G Receiver-operating characteristic (ROC) plots: a fundamental evaluation tool in clinical medicine Clin Chem 1993 39 561 577 8472349

[B17] Cauchi S Meyre D Durand E Proença C Marre M Hadjadj S Choquet H De Graeve F Gaget S Allegaert F Delplanque J Permutt MA Wasson J Blech I Charpentier G Balkau B Vergnaud AC Czernichow S Patsch W Chikri M Glaser B Sladek R Froguel P Post genome-wide association studies of novel genes associated with type 2 diabetes show gene-gene interaction and high predictive value PLoS ONE 2008 3 e2031 10.1371/journal.pone.0002031 18461161 PMC2346547

[B18] Spector TD MacGregor AJ Risk factors for osteoarthritis: genetics Osteoarthritis Cartilage 2004 12 Suppl A S39 S44 10.1016/j.joca.2003.09.005 14698640

[B19] Yang Q Khoury MJ Friedman J Little J Flanders WD How many genes underlie the occurrence of common complex diseases in the population? Int J Epidemiol 2005 34 1129 1137 10.1093/ije/dyi130 16043441

[B20] Valdes AM Doherty M Spector TD The additive effect of individual genes in predicting risk of knee osteoarthritis Ann Rheum Dis 2008 67 124 127 10.1136/ard.2007.075838 17704066

[B21] Sanna S Jackson AU Nagaraja R Willer CJ Chen WM Bonnycastle LL Shen H Timpson N Lettre G Usala G Chines PS Stringham HM Scott LJ Dei M Lai S Albai G Crisponi L Naitza S Doheny KF Pugh EW Ben-Shlomo Y Ebrahim S Lawlor DA Bergman RN Watanabe RM Uda M Tuomilehto J Coresh J Hirschhorn JN Shuldiner AR Common variants in the GDF5-UQCC region are associated with variation in human height Nat Genet 2008 40 198 203 10.1038/ng.74 18193045 PMC2914680

[B22] Weedon MN McCarthy MI Hitman G Walker M Groves CJ Zeggini E Rayner NW Shields B Owen KR Hattersley AT Frayling TM Combining information from common type 2 diabetes risk polymorphisms improves disease prediction PLoS Med 2006 3 e374 10.1371/journal.pmed.0030374 17020404 PMC1584415

[B23] Miyake K Yang W Hara K Yasuda K Horikawa Y Osawa H Furuta H Ng MC Hirota Y Mori H Ido K Yamagata K Hinokio Y Oka Y Iwasaki N Iwamoto Y Yamada Y Seino Y Maegawa H Kashiwagi A Wang HY Tanahashi T Nakamura N Takeda J Maeda E Yamamoto K Tokunaga K Ma RC So WY Chan JC Construction of a prediction model for type 2 diabetes mellitus in the Japanese population based on 11 genes with strong evidence of the association J Hum Genet 2009 54 236 241 10.1038/jhg.2009.17 19247372

